# Impaired dopamine metabolism is linked to fatigability in mice and fatigue in Parkinson’s disease patients

**DOI:** 10.1093/braincomms/fcab116

**Published:** 2021-06-08

**Authors:** Débora da Luz Scheffer, Fernando Cini Freitas, Aderbal Silva Aguiar Jr, Catherine Ward, Luiz Guilherme Antonacci Guglielmo, Rui Daniel Prediger, Shane J F Cronin, Roger Walz, Nick A Andrews, Alexandra Latini

**Affiliations:** 1 LABOX, Departamento de Bioquímica, Centro de Ciências Biológicas, Universidade Federal de Santa Catarina, Florianópolis, SC 88040-900, Brazil; 2 Graduate Program in Medical Sciences, University Hospital, Universidade Federal de Santa Catarina, Florianópolis, SC 88040-900, Brazil; 3 Neurology Division, Hospital Governador Celso Ramos, Florianópolis, SC 88015-270, Brazil; 4 Kirby Neurobiology Center, Boston Children’s Hospital and Department of Neurobiology, Harvard Medical School, Boston, MA 02115, USA; 5 Departamento de Educação Física, Centro de Desportos, Universidade Federal de Santa Catarina, Florianópolis, SC 88040-900, Brazil; 6 Departamento de Farmacologia, Centro de Ciências Biológicas, Universidade Federal de Santa Catarina, Florianópolis, SC 88040-900, Brazil; 7 Institute of Molecular Biotechnology of the Austrian Academy of Sciences, A-1090 Vienna, Austria; 8 Center for Applied Neuroscience, University Hospital, Universidade Federal de Santa Catarina, Florianópolis, SC 88040-900, Brazil; 9 Neurology Division, Departament of Internal Medicine, University Hospital, Universidade Federal de Santa Catarina, Florianópolis, SC 88040-900, Brazil; 10 The Salk in Institute for Biological Studies, La Jolla, CA 92037, USA

**Keywords:** fatigue, Parkinson’s disease, reserpine, dopamine, physical activity

## Abstract

Fatigue is a common symptom of Parkinson’s disease that compromises significantly the patients’ quality of life. Despite that, fatigue has been under-recognized as symptom, its pathophysiology remains poorly understood, and there is no adequate treatment so far. Parkinson’s disease is characterized by the progressive loss of midbrain dopaminergic neurons, eliciting the classical motor symptoms including slowing of movements, muscular rigidity and resting tremor. The dopamine synthesis is mediated by the rate-limiting enzyme tyrosine hydroxylase, which requires tetrahydrobiopterin as a mandatory cofactor. Here, we showed that reserpine administration (1 mg/kg, two intraperitoneal injections with an interval of 48 h) in adult Swiss male mice (8–10 weeks; 35–45 g) provoked striatal depletion of dopamine and tetrahydrobiopterin, and intolerance to exercise. The poor exercise performance of reserpinized mice was not influenced by emotional or anhedonic factors, mechanical nociceptive thresholds, electrocardiogram pattern alterations or muscle-impaired bioenergetics. The administration of levodopa (100 mg/kg; i.p.) plus benserazide (50 mg/kg; i.p.) rescued reserpine-induced fatigability-like symptoms and restored striatal dopamine and tetrahydrobiopterin levels. Remarkably, it was observed, for the first time, that impaired blood dopamine metabolism inversely and idependently correlated with fatigue scores in eighteen idiopathic Parkinson’s disease patients (male *n *=* *13; female *n *=* *5; age 61.3 ± 9.59 years). Altogether, this study provides new experimental and clinical evidence that fatigue symptoms might be caused by the impaired striatal dopaminergic neurotransmission, pointing to a central origin of fatigue in Parkinson’s disease.

## Teaser

Scheffer et al. report that the administration of levodopa restored striatal dopamine and tetrahydrobiopterin levels and rescued fatigability-like symptoms in reserpinized mice. The association of impaired dopaminergic system and fatigue symptoms was revealed by the inverse correlation of plasma 3,4-dihydroxyphenylacetic acid levels with fatigue scores in patients affected by Parkinson’s disease.

## Graphical Abstract

**Graphical Abstract:**
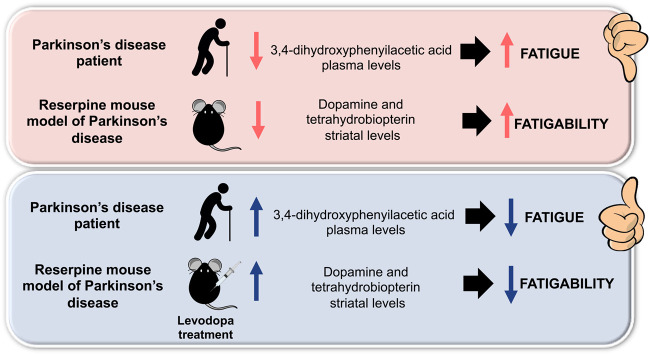

## Introduction

Parkinson’s disease is the second most common neurodegenerative disorder and it is characterized by the progressive degeneration of dopaminergic neurons in the *substantia nigra pars compacta*, leading to severe loss of dopamine in the striatum.^1^ Despite the well-known role of dopamine in the control of movements, dopamine also modulates other relevant functions including arousal, mood, motivation, learning and memory, vigilance and reward.^2^^,^^3^

Non-motor symptoms (NMS) are major determinants of quality of life in Parkinson’s disease patients, and it is now evident that these symptoms may occur across all early pre-motor and late motor stages of the disease.^4^ NMS mainly include pain, dysarthria, cognitive impairments, olfactory and gastrointestinal deficits, depression, sleep disorders, psychosis and fatigue.^4^^,^^5^ Fatigue, clinically defined as an overwhelming sense of tiredness, lack of energy and feelings of exhaustion and has a high prevalence (up to 70%) in Parkinson’s disease patients.^6–8^ Although extra-striatal dopaminergic pathways and non-dopaminergic dysfunction are thought to play a major role in fatigue development,^9^ it has also been demonstrated that levodopa is effective in reducing physical fatigue. Lou et al.^10^ demonstrated that levodopa improved physical fatigability assessed by the finger tapping and force generation tasks one hour after its administration in idiopathic Parkinson’s disease subjects.^10^ Moreover, it has been reported that blockers of dopamine transporter, such as methylphenidate and modafinil, also reduce fatigue scores supporting the involvement of dopaminergic neurotransmission dysfunction in the aetiopathology of fatigue in Parkinson’s disease.^8^^,^^11^^,^^12^

Tyrosine hydroxylase is the rate-limiting enzyme controlling the synthesis of dopamine and its activity relies on appropriate intracellular levels of its mandatory cofactor, tetrahydrobiopterin (BH4).^13–15^ Although the current understanding of the biology of BH4 in the CNS is restricted to its coenzyme activity required for the synthesis of catecholamines and nitric oxide, our group has recently demonstrated new central properties of BH4 metabolism. For instance, a single intracerebroventricular injection of BH4 enhances hippocampal synaptic plasticity, facilitating learning and memory processes in different species and strains of rodents.^16^ In consonance, it has been reported that BH4 administration in patients affected by atypical phenylketonuria, a metabolic disorder characterized by the inherited inability to convert phenylalanine into tyrosine due to BH4 deficiency, improved working memory and induced neuronal activation in the prefrontal and parietal cortex, which was demonstrated by *in vivo* metabolic imaging.^17^ In this context, it is known that dopamine depletion leads to prefrontal dysfunction which, in turn, contributes to executive function impairments often seen in Parkinson’s disease patients.^18^ In addition, reduced CSF BH4 levels have been observed in Parkinson’s disease patients^19^ and in the striatum of mice treated with 1-methyl-4-phenyl-1,2,3,6-tetrahydropyridine, a neurotoxin widely used to model of Parkinson’s disease,^20^ underlying the participation of this enzyme cofactor in the physiopathology of the disease.

Levodopa remains the most effective drug to alleviate the motor symptoms of Parkinson’s disease, including muscle rigidity, resting tremor and bradykinesia. However, the putative benefits of levodopa treatment, and the role of the dopaminergic system on fatigue symptoms remain under investigated. Here, we observed that levodopa administration attenuated fatigability-like symptoms by restoring striatal dopamine and BH4 levels in a mouse model of Parkinson’s disease induced by reserpine administration, a blocker of the vesicular monoamine transporter 2.^21^ Additionally, the translational facet of this work demonstrated an inverse correlation of the dopamine turnover with the fatigue and motor scores in idiopathic Parkinson’s disease patients. These findings indicate that dopaminergic deficiency underlies the development of fatigue symptoms in Parkinson’s disease.

## Materials and methods

### Participants

Outpatients with Parkinson’s disease from the public Movement Disorders Clinic of Santa Catarina State at the Hospital Governador Celso Ramos, in Florianópolis city, southern Brazil were included in this study. BH4, dopamine and 3,4-dihydroxyphenyilacetic acid (DOPAC) levels were quantified in a cohort of 18 patients (control group: 18 healthy controls), see Supplementary Table 1 for the demographic and clinical characteristics of patients. Diagnosis was based on the Queen Square Brain Bank according to the clinical.^22^ They were diagnosticated, examined and managed by a board-certified neurologist trained in movement disorders. Patients had bradykinesia, and at least two of the following symptoms: (i) resting tremor (frequency between 4 and 6 Hz); (ii) muscle rigidity; and (iii) postural instability not caused by visual or vestibular or cerebellar proprioceptive dysfunction. All patients’ symptoms improved substantially under dopamine agonists or levodopa treatment. The disease stage was evaluated using the Hoehn and Yahr scale.^23^ Anxiety and depressive symptoms were determined by the Hospital Anxiety and Depression scale (HADS)^24^ validated for Brazilian patients.^25^^,^^26^

Exclusion criteria for Parkinson’s disease: previous history of cerebrovascular disease, brain trauma, infection or tumour, oculogyric crisis, neuroleptic use, prolonged symptoms remissions; strictly unilateral symptoms after three years of disease, supra-nuclear palsy, cerebellar symptoms, early-onset dysautonomia or dementia, pyramidal signs, brain tumour and hydrocephalus. Parkinson’s disease symptoms were assessed using the Movement Disorder Society-sponsored revision of the Unified Parkinson’s Disease Rating Scale (UPDRS).^27^ NMS, namely apathy, sleep problems and daytime sleepiness from the UPDRS Part I were also evaluated. All patients were on their regular medication and best ‘on state’ (Table 1). Blood samples were collected immediately after the neurologic evaluation.

**Table 1:** Association between Parkinson’s Fatigue Score (PFS) and the plasma levels of dopamine, tetrahydrobiopterin (BH4), 3,4-dihydroxyphenylacetic acid (DOPAC) and the socio-demographic and clinical characteristics of patients

Predictive variables	Linear regression coefficients			*P*
	*r*	*r* ^2^	B (CI 95%)	level
Blood parameters				
Dopamine levels (nmol/l)	0.32	0.10	2.51 (−7.30 to 2.28)	0.27
BH4 levels (nmol/l)	0.40	0.11	0.55 (−0.14 to 1.24)	0.11
DOPAC levels (nmol/l)	0.58	0.34	−1.86 (−3.49 to −0.23)	**0.03**
Socio-demographic and clinical characteristics				
Age, years	0.33	0.11	−0.65 (−1.67 to 0.37)	0.19
Female	0.22	0.05	10.4 (−13.9 to 34.0)	0.38
Disease duration, years	0.28	0.08	−0.99 (−2.84 to 0.86)	0.27
Hoehn and Yard stage	0.10	0.01	1.26 (−0.03 to 8.56)	0.71
Daily dose Levodopa (mg/kg)	0.04	0.00	0.08 (−1.01 to 1.25)	0.88
MOCA score	0.06	0.00	0.16 (−1.42 to 1.75)	0.83
Education (years)	0.03	0.00	0.11 (−2.15 to 2.37)	0.92
HADS depression score	0.56	0.31	2.18 (0.41 to 3.96)	**0.02**
HADS anxiety score	0.44	0.19	2.10 (−0.25 to 4.44)	**0.08**
Body mass index (kg/m^2^)	0.78	0.62	2.60 (1.30 to 3.88)	**0.001**
UPDRS—Part I				
Apathy	0.05	0.003	1.04 (−9.88 to 11.96)	0.84
Sleep problems	0.22	0.05	0.57 (−0.86 to 1.99)	0.41
Daytime sleepiness	0.48	0.24	10.51 (0.09 to 20.94)	**0.05**
UPDRS—Part III (motor evaluation)	0.35	0.12	0.41 (−0.29 to 1.12)	0.20
Final model^b^	0.95	0.91		0.005
Constant			22.57 (18.58 to 64.29)	0.003
IMC			0.66 (−1.10 to 2.41)	0.38
UPDRS-III score			0.62 (0.09 to 1.12)	**0.02**
DOPAC levels (nmol/l)			−2.27 (−3.57 to −0.97)	**0.003**

a Univariate analysis by linear regressions.

b Final Model of multiple linear regressions showing the independent association between PFS score variation and the UPDRS-III score and the plasmatic levels of DOPAC. Only these two variables remained significant in the final model.

The control group was composed of healthy donors without neurologic or psychiatric diseases, matched by age and gender. Healthy donors were people accompanying patients who were seen in other clinics at the hospital, elderly individuals enrolled in continuing education (NETI Group), and professors and researchers working at Universidade Federal de Santa Catarina, Florianópolis, Brazil. Exclusion criteria for healthy donors: (i) use of anti-inflammatory in the last 3 weeks; (ii) acute disease, transmissible or not, in the last 60 days; (iii) active or ‘in remission’ cancer; and (iv) hepatic or renal insufficiency.

The project was approved by the Ethics Committee (CEP-HGCR 2009-26) and followed the guidelines of the Code of Ethics of the World Medical Association (Declaration of Helsinki; Ref World Medical Association; World Medical Association Declaration of Helsinki: ethical principles for medical research involving human subjects. JAMA. 2013; 310:2191-4). A signed informed consent was obtained from all participants.

### Parkinson Fatigue Scale

The fatigue symptoms of Parkinson’s disease patients were assessed by the validated version of Parkinson Fatigue Scale (PFS-16) for Brazilian individuals.^28^ The PFS-16 is a self-report questionnaire of 16 questions used to screen for the presence and severity of fatigue.^29^ For each question, the patients chose how much they agreed or disagreed with the statements. The higher the scores, the more severe the fatigue.

### Cognitive function

Cognitive function was evaluated by the Montreal Cognitive Assessment (MOCA).^30^ The validated version adapted to Brazilians^31^ was applied to assess attention, executive functions, memory, language, visuoconstructional skills and orientation. MOCA is 30 points score scale and the higher the scores, the better the cognitive function.

### Anxiety and depressive symptoms

The level of anxiety and depressive symptoms of Parkinson’s disease patients were assessed by applying the adapted version for Brazilians of the psychometric scale HADS.^24–26^ HADS is a widely used 14-item scale divided into subscales with seven items for anxiety and seven items for depression, resulting in a final score from 0 to 21 in each subscale. Higher scores indicate more symptoms of anxiety and depression.

### Blood human collection

After applying the fatigue questionnaire, 4 ml blood sample was drawn from the median cubital vein and distributed into sterile vacutainer tubes containing 10% EDTA. The blood was immediately centrifuged at 400 × *g*, for 10 min at room temperature. Plasma was collected for catecholamines analyses and frozen and stored −86°C, until analysis.

### Animals

Male Swiss mice (8–10 weeks, 35–45 g) from the animal facility of the Center for Biological Sciences, Universidade Federal de Santa Catarina, Florianopolis, Brazil, and C57Bl6j male mice (10–12 weeks, 23–28 g) obtained from Jackson Laboratory, CT, USA, were kept in a controlled environment (22°C ± 1°C, 12 h light/dark cycle, lights ON at 7:00 a.m.) with water and food *ad libitum*. The experimental protocols were approved by the Ethics Committee for Animal Research (PP00760/CEUA) of the Universidade Federal de Santa Catarina Florianopolis, Brazil, and by the Boston Children’s Hospital Animal Care and Use Committee (13-01-2334). Experiments were carried out in accordance with the European Communities Council Directive of 24 November 1986 (86/609/EEC).

### Cell culture

L6 cells, a myoblast cell line representative of rat skeletal muscle, were purchased from the Cell Bank of Rio de Janeiro (Rio de Janeiro, Brazil). The cells were seeded in flasks and cultured in Dulbecco’s modified Eagle’s medium (DMEM), pH 7.4, containing 10% foetal bovine serum, sterile antimycotic solution 100× penicillin 100 IU/ml, streptomycin 0.1 mg/ml and amphotericin 0.25 μg/ml, d-glucose 5.5 mM, 2% glutamine, 0.22% NaHCO_3_, and 0.47% HEPES in a 95% O_2_ and 5% CO_2_ humidified atmosphere, at 37°C. After confluence, L6 myotubes were maintained *in vitro* with changes of medium every 48 h prior to the experiments.

### Striatal dopamine depletion induced by reserpine

**Figure 1:**
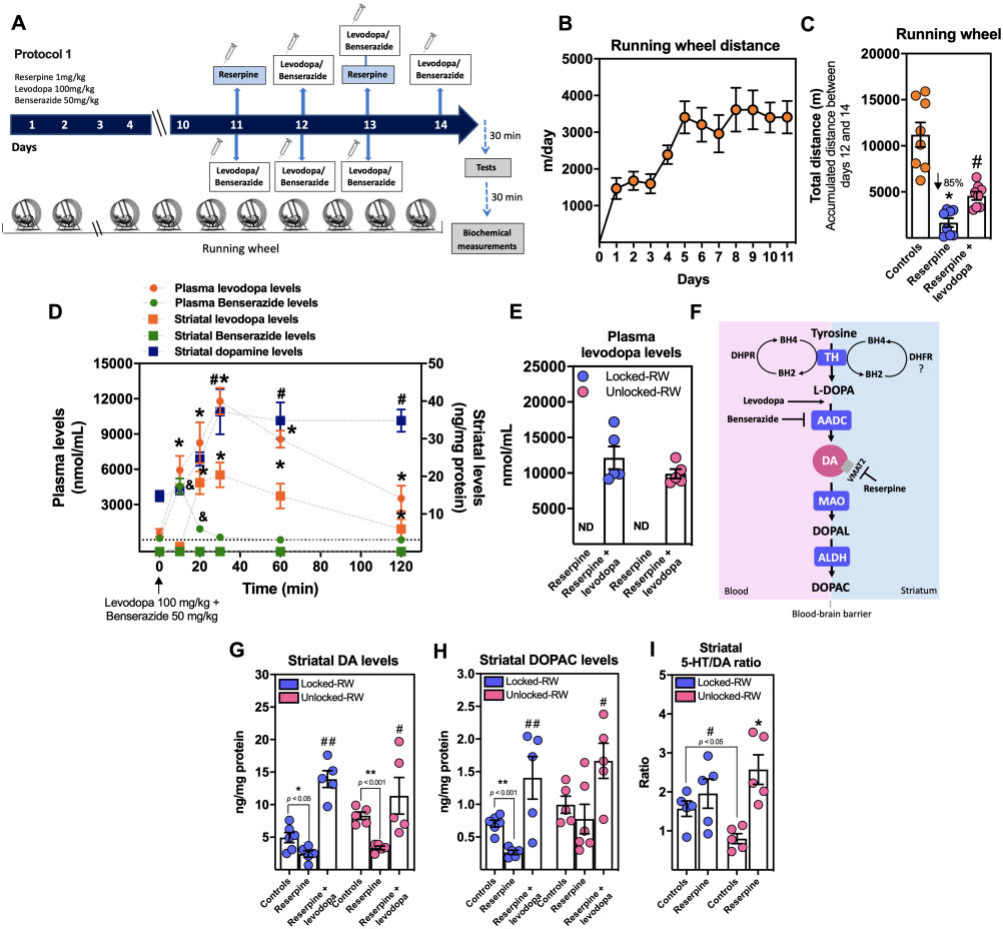
**Effect of levodopa treatment on physical activity and dopaminergic system in reserpinized mice.** (**A**) Protocol design 1. For details, see Materials and Methods. (**B**) Daily travelled distance in the running wheel (RW) for Swiss mice submitted to voluntary exercise for 11 days (*n *=* *8 mice/group). (**C**) Accumulated distance travelled in the RW between days 12 and 14 (*n *=* *8 mice/group; one-way ANOVA followed by the Dunnett’s test; *P <* *0.05 vs. controls and ^#^*P *<* *0.05 vs. reserpine-treated mice). (**D**) Pharmacokinetics of plasma levodopa levels (orange circle symbols), plasma benserazide levels (green circle symbols), striatal levodopa levels (orange square symbols), striatal benserazide levels (green square symbols), striatal dopamine levels (blue square symbols) after a single dose of levodopa/benserazide (100/50 mg/kg; i.p; Swiss mice; *n *=* *5 mice/time; repeated measure one-way ANOVA followed by the Bonferroni test; levodopa: *P <* *0.001 vs. time 0, benserazide: ^&^*P *<* *0.001 vs. time 0 and dopamine: ^#^*P *<* *0.05 vs. time 0). (**E**) Plasma levodopa levels (Swiss mice; *n *=* *5 mice/group; locked-RW and unlocked-RW; ND: undetectable). (**F**) Local of action of levodopa, benserazide and reserpine in the dopamine synthesis and vesicular storage (AADC, aromatic l-amino acid decarboxylase; BH2, dihydrobiopterin; BH4, tetrahydrobiopterin; DA, dopamine; DHFR, dihydrofolate reductase; DHPR, dihydropteridin reductase; DOPAC, 3,4-dihydroxyphenylacetic acid; DOPAL, 3,4-dihydroxyphenylacetaldeyde; MAO, monoamine oxidase; TH, tyrosine hydroxylase; VMAT2, vesicular monoamine transporter 2). (**G**) Striatal dopamine levels (Swiss mice; *n *=* *5 mice/group; one-way ANOVA followed by the Bonferroni test; ^#^*P *<* *0.05; ^##^*P *<* *0.01 vs. reserpine-treated mice and Student’s *t*-test; ^*^P <* *0.05, ^**^P <* *0.001 vs. controls; locked-RW and unlocked-RW). (**H**) Striatal 3,4-dihydroxyphenylacetic acid (DOPAC) levels (Swiss mice; *n *=* *5 mice/group; one-way ANOVA followed by the Bonferroni test; ^#^*P *<* *0.05; ^##^*P *<* *0.01 vs. reserpine-treated mice and Student’s *t*-test; ^**^P <* *0.001 vs. controls; locked-RW and unlocked-RW). (**I**) Striatal serotonin (5HT)/dopamine (DA) ratio (Swiss mice; *n *=* *5 mice/group; two-way ANOVA followed by the Bonferroni test; *P <* *0.05 vs. controls and Student’s *t*-test; ^#^*P *<* *0.05 vs. unlocked-RW; locked-RW and unlocked-RW). Results are presented as mean ± SEM.

**Figure 2:**
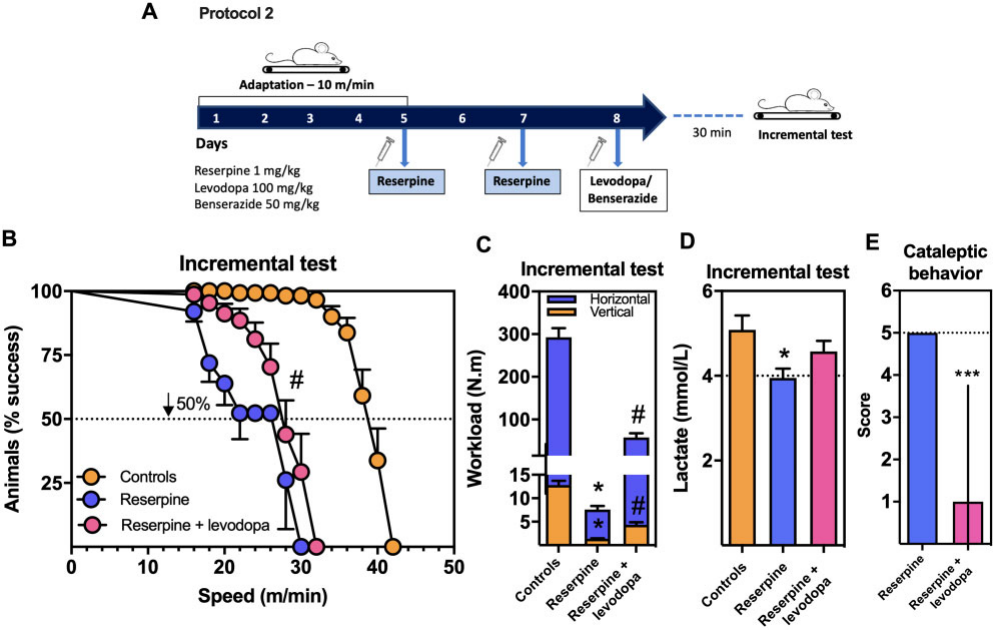
**Effect of levodopa treatment on physical performance and cataleptic scores in reserpinized mice.** (**A**) Protocol design 2. For details, see Materials and Methods. (**B**) Mouse physical performance on treadmill exhaustion test [Swiss mice; *n *=* *15 mice/group; Log-rank (Mantel–Cox); ^#^*P *<* *0.0001 vs. reserpine-treated mice]. (**C**) Vertical and horizontal workloads during exhaustion test (Swiss mice; *n *=* *15 mice/group; one-way ANOVA followed by the Bonferroni test; *P <* *0.05 vs. controls and ^#^*P *<* *0.05 vs. reserpine-treated mice). (**D**) Blood lactate levels (Swiss mice; *n *=* *15 mice/group; one-way ANOVA followed by the Bonferroni test; *P <* *0.05 vs. controls). (**E**) Cataleptic scores (Swiss mice; *n *=* *20 mice/group; Student’s *t*-test. ***P <* *0.001 vs. reserpine-treated mice). Results are presented as mean ± SEM. except for the cataleptic test, which are presented as median ± interquartile range.

Dopamine levels were pharmacologically depleted by two i.p. injections of reserpine (1 mg/kg) separated by an interval of 48 h, as shown in Figs 1A and 2A. Controls received 0.1% glacial acetic acid (vehicle).^32^^,^^33^ Reserpine inhibits the vesicular monoamine transporter 2 leading to a loss of storage capacity of monoamines in synaptic vesicles, causing depletion in the brain and peripheral monoamines levels. For additional drugs detail, see the Supplementary material.

### Physical activity protocols

#### Running wheel protocol

To monitor daily running distances, mice were housed in individual cages (27 × 18 × 13 cm) with free access to locked (non-exercised mice) or unlocked (exercised) functional running wheels (4½″, Super Pet, USA). Locked running wheels were used to mitigate the bias of environmental enrichment. Electronic counters were connected to the running wheels to quantify the daily travelled distance.^34^

#### Exhaustion test protocol

The incremental loading test was performed as previously reported by our group.^35^ For additional details, see the Supplementary material.

### Behavioural paradigms

#### Protocol 1

The animals were randomized and the effects of reserpine alone or combined with levodopa/benserazide on running wheel performance were investigated in mice submitted to voluntary exercise according to Fig. 1A. Pharmacological challenges were performed in the last four days (days 11–14) of voluntary exercise. Mice received two injections of vehicle or reserpine (1 mg/kg, 48 h interval) on days 11 and 13. Then, the animals were treated with levodopa/benserazide (100/50 mg/kg, i.p.). After the first reserpine treatment, mice received levodopa/benserazide with 12 h intervals during four consecutive days (days 11–14). Controls were always treated with appropriate vehicles. The distance travelled on the running wheel was monitored for 14 days. Thirty minutes after the last levodopa administration, cataleptic scores and depressive-like behaviours were assessed. Afterwards, mice were euthanized and the striatum dissected for catecholamine quantitation.

#### Protocol 2

Mice were randomized and submitted to the exhaustion test protocol in order to assess the effect of levodopa/benserazide treatment on the animal’s maximal treadmill performance after reserpine administration. Pharmacological challenges were performed after five days of habituation to the treadmill. Mice received two injections of vehicle or reserpine (1 mg/kg) on days 5 and 7. A single dose of levodopa/benserazide (100/50 mg/kg, i.p.) was administered 30 min before the treadmill challenge. Controls were always treated with appropriate vehicles (Fig. 2A).

### Behavioural measurements

Catalepsy, forced swimming, splash, von Frey tests and ECG profile were scored by the same rater in an observation room where the mice were habituated for at least 1 h. The measurements were blinded with regard to the treatment.

#### Catalepsy test

The forelimbs were placed on a bar (Ø 3 mm, 4.5 high, 10 cm wide) and the cataleptic time of fore/hindlimbs was independently scored as previously described.^36^

#### Forced swimming test

Depressive-like symptoms were assessed on the forced swimming test and body immobility was scored during 5 min, as previously reported.^37^^,^^38^ For additional details, see the Supplementary material.

#### Splash test

The self-care and motivational behaviours were assessed by measuring the time spent in grooming behaviour, as previously reported.^39^ For additional details, see the Supplementary material (Videos 1 and 2).

#### Von Frey test

The threshold for mechanical nociception was determined with a graded series of eight von Frey filaments, as previously reported.^40^^,^^41^ For additional details, see the Supplementary material.

#### ECG profile

Heart's rhythm and activity was determined by assessing the ECG (Mouse Specifics, MA, USA). The recording rate interval and heart rate were calculated automatically by the software. For additional details, see the Supplementary material.

### Biochemical measurements

#### Quantitation of l-3,4-dihydroxyphenylalanine, dopamine, DOPAC and serotonin levels

Monoamines and their metabolites were measured in the striatum and/or the blood by high performance liquid chromatography (HPLC) followed by electrochemical detection, following an established protocol.^35^ For additional details, see the Supplementary material.

#### Quantitation of BH4 levels

Striatal BH4 levels were determined by HPLC coupled with electrochemical detector, as previously described with some modifications.^41^^,^^42^ For additional details, see the Supplementary material.

#### Quantitation of sepiapterin levels

Serum sepiapterin levels were performed by coupling to the HPLC system a multi-wavelength fluorescence detector.^43^ For additional details, see the Supplementary material.

#### Gene expression analysis by quantitative real-time PCR

Total RNA was isolated from striatum samples by using the TRIzol^®^/chloroform/isopropanol method as previously described.^16^ Primers used for gene expression analysis are described in Supplementary Table 2. For additional details, see the Supplementary material.

#### Cellular viability assay

MTT (3[4,5-dimethylthiazol-2-yl]-2,5-diphenyltetrazolium bromide) assay was used to evaluate cellular viability in L6 myotubes.^44^ For additional details, see the Supplementary material.

#### Quantitation of lactate levels

Blood lactate levels were analysed using a specific analyzer (YSL 2700, CA, USA). The results were calculated as mmol/L.

#### Measurement of mitochondrial respiration

Oxygen consumption was measured in L6 myotubes by high-resolution respirometry using the Oroboros^®^ oxygraph, as previously described by our group.^45^ For additional details, see the Supplementary material.

#### Protein determination

Protein concentration was determined according to the Lowry method using bovine serum albumin as standard.^46^

### Statistical analysis

Results are presented as mean ± SEM. Data generated from animal studies were statistically analysed by applying Student’s *t*-test one-tailed (dopamine and DOPAC levels, serotonin to dopamine ratio, cataleptic score) and two-tailed (mitochondrial respiration); and one-way ANOVA (distance travelled, workload, dopamine, DOPAC, BH4 and sepiapterin levels, serotonin to dopamine ratio, forced swimming test and splash test, von Frey measurements, ECG profile, recording rate interval and heart rate, lactate levels, MTT reduction, levodopa and BH4 curve, BH4 pathway gene expression). The non-parametric cataleptic data are presented as median ± interquartile range. The results from the exhaustion test are presented in percentage of animals that successfully performed the task at specific speeds. In this case, group differences were examined by applying Log-rank (Mantel–Cox). The accepted level of significance for the tests was *P *<* *0.05.

Data generated from Parkinson’s disease patients were first analysed by applying descriptive statistics (demographic, clinical and laboratory data), and then a univariate analysis was performed by linear regression to identify whether blood dopamine-related metabolites (dopamine, BH4 and DOPAC), socio-demographic and clinical variables were associated with fatigue scores. The level of significance for this analysis was *P *≤* *0.20. Since it was observed a collinearity between HADS anxiety and HADS depression, only the latter was included in the multiple regression analysis. No collinearities were observed among the other investigated scales. BH4 levels, DOPAC levels, HADS depression, daytime sleepiness (from the UPDRS I score), motor symptoms (UPDRS III full score) and body mass index were included in a multiple linear regression analysis to identify independent predictors for fatigue scores variation. The level of association was determined by the ‘*r*’ and ‘*r*^2^’ coefficients, and the *P* level ≤ 0.05 was considered significant. Considering the clinical and biological plausibility of the investigated variables, and to avoid a type II error, no corrections for multiple comparisons were applied. Statistics and all graphs were performed by using SPSS^®^ Statistics 20.0 and GraphPad Prism 7^®^, respectively.

### Data availability

The data that support the findings of this study are available from the corresponding author, upon request.

## Results

### Striatal dopamine depletion impaired exercise performance in reserpine-treated mice

Fatigability-like symptoms, measured as a decline in physical activity, were investigated in reserpinized mice with impaired striatal dopaminergic neurotransmission. Running wheels have been previously characterized as viable tools for measuring exercise intolerance behaviour in mice, with the advantage that they do not interfere with the normal voluntary activity of the rodent.^47^ Mice were kept in individual cages with a functional running wheel for 14 days (protocol 1, see Materials and Methods; Fig. 1A). Figure 1B shows that naïve mice submitted to voluntary exercise reached a maximal travelled distance of 3505 ± 345 m/day on day 8, and that this level was maintained until day 11, when mice were submitted to protocol 1. As expected, the administration of reserpine (on days 12 and 14) impaired motor activity as shown by the significant reduction in the accumulated travelled distance during days 11–14 (up to 85% reduction) compared to controls [*F*(2,21) = 32.40; *P *<* *0.05] (Fig. 1C). Exercise fatigability was also confirmed in a non-voluntary setting, by submitting the animals to a treadmill fatigability/exhaustion test^48^ (protocol 2, see Materials and Methods; Fig. 2A). Reserpine-treated mice also showed impaired performance (up to 50% compared to controls), [χ^2^_(2)_ = 123.10; *P *<* *0.001]; Fig. 2B) under this paradigm with significant reduced horizontal [*F*(2,48) = 103.30; *P *<* *0.05] and vertical [*F*(2,48) = 81.81; *P *<* *0.05] exercise workloads (Fig. 2C). Lower lactate levels were observed in reserpine-treated mice [*F*(2,42) = 4.11; *P *<* *0.05] (Fig. 2D), which are in agreement with the observed reduced exercise workload (Fig. 2C). The reserpine-induced performance fatigability was biochemically characterized by decreased levels of dopamine [*t*_(8)_ = 2.97; *P *<* *0.05] (Fig. 1G) and DOPAC [*t*_(8)_ = 5.89; *P *<* *0.001] (Fig. 1H) in the mouse striatum, which were reverted by the administration of levodopa/benserazide [*F*(5,23) = 6.15; *P *<* *0.05] (Fig. 1G); [*F*(5,24) = 5.24; *P *<* *0.05] (Fig. 1H). Furthermore, the striatal serotonin to dopamine ratio was significantly increased in the striatum of reserpine-injected animals [*F*(3,12) = 10.74; *P *<* *0.05], while the lowest ratio was observed in exercised mice from the control group which showed the highest exercise performance [*t*_(8)_ = 3.12; *P *<* *0.05] (Fig. 1I).

The administration of the drugs used to rescue exercise performance was based on their plasma and striatum pharmacokinetics as shown in Fig. 1D. A single dose of levodopa (100 mg/kg) generated the highest plasma and striatal levels 30 min after the administration [*F*(5,20) = 58.05; *P *<* *0.001]. These plasma and striatal peaks occurred along with the greatest striatal conversion of levodopa into dopamine [*F*(5,20) = 6.84; *P *<* *0.05]. On the other hand, benserazide levels increased in plasma 10 min after its administration, and decreased rapidly over the time [*F*(3,12) = 41.71; *P *<* *0.05]. As Fig. 1F shows, the administration of levodopa reaches the brain increasing dopamine availability, and it can only be detected in plasma from treated animals (Fig. 1E). Brain dopamine levels are determined by the action of tyrosine hydroxylase that reduces tyrosine into l-3,4-dihydroxyphenylalanine (l-DOPA) under the presence of the mandatory cofactor and electron donor, BH4. Subsequently, l-DOPA is decarboxylated by the aromatic l-amino acid decarboxylase into dopamine. Dihydrobiopterin is reduced back to BH4 by the dihydropteridin reductase mainly in the liver and possibly by the dihydrofolate reductase in the brain.^13–15^ Once dopamine is formed, the catecholamine is vesicularized via the vesicular monoamine transporter 2, which is blocked by reserpine. Once in the cytosol, dopamine is oxidized by the enzyme monoamine oxidase to 3,4-dihydroxyphenylacetaldeyde, which in turn is metabolized to form DOPAC by the action of the enzyme aldehyde dehydrogenase.^49^ Benserazide inhibits aromatic l-amino acid decarboxylase activity avoiding the formation of dopamine exclusively in the periphery, since the drug does not cross the blood-brain barrier (no benserazide levels were observed in the striatum; Fig.1D). Finally, in order to confirm that the administration of levodopa also prevented the development of Parkinson’s disease-like motor symptoms, the cataleptic score was assessed as an index of muscular rigidity. Figure 2E shows that catalepsy was significantly reduced by levodopa/benserazide treatment [*t*_(38)_ = 7.38; *P *<* *0.001].

### Reserpine-induced striatal dopamine depletion was rescued by reestablishing BH4 levels

**Figure 3:**
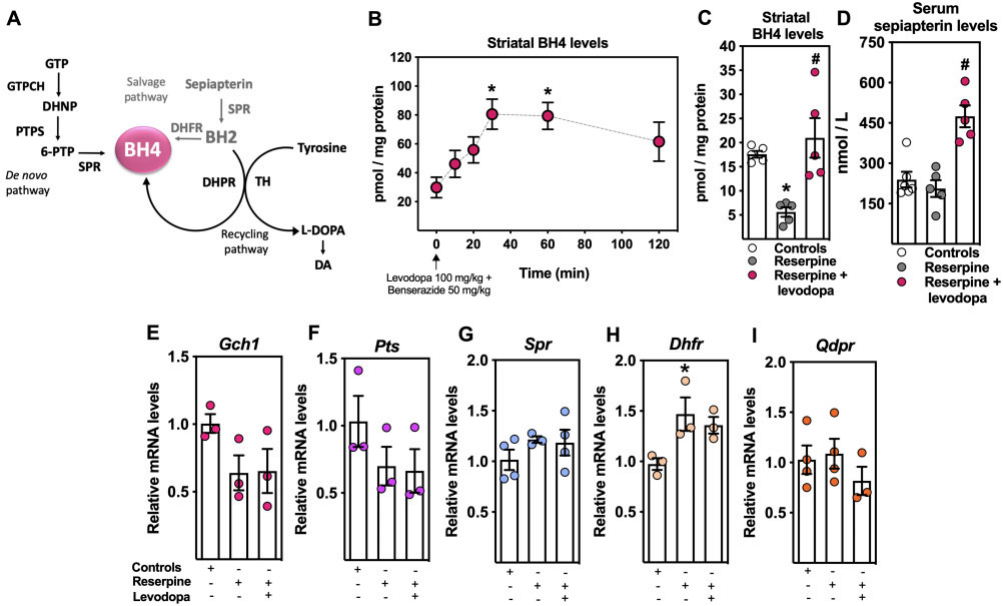
**Effect of levodopa treatment on tetrahydrobiopterin (BH4) metabolism in reserpinized mice.** (**A**) Schematic representation of the BH4 metabolism. Physiological intracellular levels of BH4 are tightly tuned by the combined action of three metabolic routes, namely the *de novo* synthesis, and the recycling and salvage pathways. The *de novo* synthesis of BH4 occurs via the successive action of the following three enzymes: guanosine triphosphate cyclohydrolase I (GTPCH), 6-pyruvoyl tetrahydropterin synthase (PTPS) and sepiapterin reductase (SPR).^13^^,^^15^ The salvage pathway rescues metabolic intermediates of the *de novo* pathway to produce sepiapterin and dihydrobiopterin (BH2), which is further reduced to BH4 by dihydrofolate reductase (DHFR). Finally, once BH4 is oxidized to BH2, during its role as an enzyme cofactor, a NADH-dependent reaction will efficiently recycle it back to BH4, maintaining BH4 appropriate levels. Intracellular BH4 is known to act as a mandatory cofactor for all amino acid hydroxylases including phenylalanine, tryptophan and tyrosine hydroxylases (TH). TH is the rate-limiting enzyme controlling the synthesis of dopamine (DA). (**B**) Pharmacokinetics of BH4 in the mouse striatum after a single dose of levodopa/benserazide (100/50 mg/kg; i.p; Swiss mice; *n *=* *4–5 mice/time; repeated measure one-way ANOVA followed by the Bonferroni test; *P <* *0.05 vs. time 0). (**C**) Striatal BH4 levels (Swiss mice; *n *=* *5 mice/group; one-way ANOVA followed by the Bonferroni test; *P <* *0.05 vs. controls and ^#^*P *<* *0.05 vs. reserpine-treated mice). (**D**) Serum sepiapterin levels (Swiss mice; *n *=* *5 mice/group; one-way ANOVA followed by the Bonferroni test; ^#^*P *<* *0.05 vs. reserpine-treated mice). Gene expression of the BH4 biosynthetic enzymes (**E**) *Gch1*, (**F**) *Pts*, (**G**) *Spr*, (**H**) *Dhfr* and (**I**) *Qdpr* analysed by qPCR (Swiss mice; *n *=* *3–4 mice/group; one-way ANOVA followed by the Tukey test; *P <* *0.05 vs. controls). Results are presented as mean ± SEM.

Figure 3C shows that dopamine depletion induced by reserpine occurred in parallel with compromised striatal BH4 production; and both dopamine and BH4 levels were rescued by levodopa treatment [*F*(2,11) =9.71; *P *<* *0.05] (Figs 1G and 3C). In addition, it can be observed in Fig. 3B that levodopa plus benserazide treatment increased the levels of BH4 in the striatum, reaching the highest level 30 min after the drugs’ injection, and remained elevated up to 60 min [*F*(5,20) = 3.46; *P *<* *0.05]. The restoration of dopamine striatal levels appears to involve the modulation of the metabolic route known as the BH4 salvage pathway (Fig. 3A), since the key metabolic intermediate sepiapterin was significantly increased in the serum of levodopa-treated animals [*F*(2,11) = 15.47; *P *<* *0.05] (Fig. 3D). The levodopa treatment also prevented the reserpine-induced upregulation of *Dhfr*, a key enzyme in the BH4 salvage pathway [*F*(2,6) = 5.36; *P *<* *0.05] (Fig. 3H). The expression of the genes that codify for the BH4 biosynthetic enzymes, guanosine triphosphate cyclohydrolase I (*Gch1*), 6-pyruvoyl tetrahydropterin synthase (*Pts*), sepiapterin reductase (*Spr*) and dihydropteridine reductase (*Qdpr*), remained unchanged (Fig. 3E–G, I).

### No emotional, nociceptive, physiology or metabolic factors explained fatigability in reserpine-treated mice

**Figure 4:**
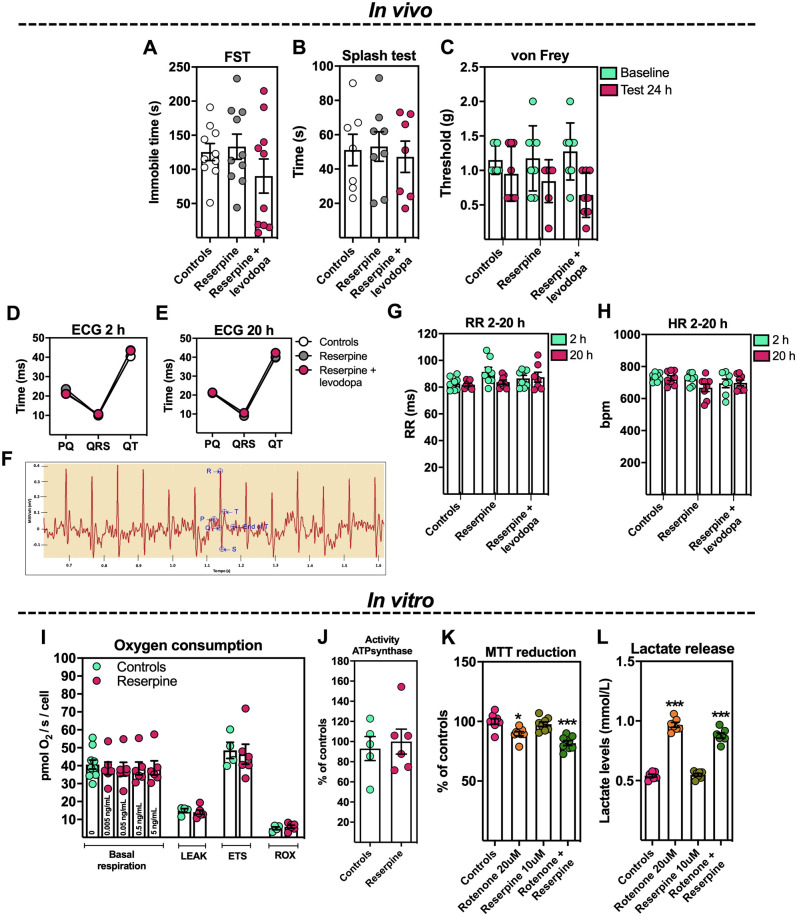
**The effect of reserpine administration on mouse behaviour and cardiovascular functions and the *in vitro* effect of the alkaloid on bioenergetics in L6 myotubes.** (**A**) Forced swimming test (FST; Swiss mice; *n *=* *10 mice/group; one-way ANOVA). (**B**) Splash test (C57Bl6j mice; *n *=* *7–8 mice/group; one-way ANOVA). (**C**) von Frey test (C57Bl6j mice; *n *=* *8 mice/group; one-way ANOVA). (**D**) Electrocardiogram (ECG) 2 h after reserpine administration (C57Bl6j mice; *n *=* *8 mice/group; one-way ANOVA), and (**E–F**) ECG 20 h after reserpine injection (C57Bl6j mice; *n *=* *8 mice/group; one-way ANOVA). (**G**) Recording rate 2 h and 20 h after reserpine injection (recording rate interval-RR; C57Bl6j mice; *n *=* *8 mice/group; one-way ANOVA). (**H**) Heart rate 2 h and 20 h after reserpine injection (heart rate-HR; C57Bl6j mice; *n *=* *8 mice/group; one-way ANOVA). (**I**) Mitochondrial oxygen consumption in L6 muscle cells (*n *=* *4–6; Student’s *t*-test; ETS: maximal mitochondrial electron transfer activity; LEAK: electron flow coupled to proton pumping to compensate for proton leaks; ROX: residual oxygen consumption). (**J**) Contribution of ATP synthase activity to basal oxygen consumption (basal respiration) in L6 muscle cells (*n *=* *5–6; Student’s *t*-test). (**K**) Cell viability in L6 muscle cells (*n *=* *6; two-way ANOVA followed by the Bonferroni test; *P <* *0.05, ***P <* *0.0001 vs. controls). (**L**) Lactate release in L6 muscle cells (*n *=* *6; two-way ANOVA followed by the Bonferroni test; ***P <* *0.0001 vs. controls). Rotenone (20 μM) was used as positive control. Results are presented as mean ± SEM. **Video 1.** Representative video of one mouse from the control group during the splash test (Fig. 4B). **Video 2.** Representative video of one mouse from the reserpine group during the splash test (Fig. 4B).

In order to rule out the involvement of depressive-like symptoms, reduced nociceptive threshold, altered autonomic nervous system or energy deficiency in the induction of fatigability, reserpine-treated mice were submitted to a set of behavioral tests. Figure 4A and B shows that the impaired exercised performance induced by dopamine and BH4 deficiency was not due to anhedonic, motivational or depressive-like symptoms since no differences were observed between control and treated animals in the forced-swimming test (Fig. 4A) nor in the splash test (Fig. 4B; Videos 1 and 2). Moreover, no changes were observed in mechanical nociceptive thresholds (Fig. 4C) or in the cardiovascular physiology as evaluated by ECG profiling (Fig. 4D–F), as well as the recording rate interval and heart rate, 2 h or 20 h after reserpine administration (Fig. 4G and H, respectively). These data suggest that reserpine administration did not modify nociception or impair heart physiology by inducing hypotension (due to catecholamine depletion) in the induction of fatigability. Importantly, levodopa treatment also did not change any of these behavioral parameters (Fig. 4A–H). Finally, Fig. 4I–L shows that increasing concentration of reserpine (0.005–5 ng/ml) did not compromise the bioenergetics (Fig. 4I, J), including the production of lactate (Fig. 4L); [*F*(3,21) = 76.71; *P *<* *0.0001], or the cellular viability (Fig. 4K) of L6 myotubes.

### Fatigue was independently associated with reduced DOPAC blood levels in Parkinson’s disease patients

In order to translate the experimental findings to a clinical context, the association between demographics, clinical and biochemical data with fatigue scores was determined in 18 patients with idiopathic Parkinson’s disease. The univariate analysis presented in the Table 1 shows a significant and negative association between blood DOPAC levels (*r *=* *0.58; *P < *0.03) and PFS-16 scores, a significant and positive association between fatigue symptoms and HADS depression scores (*r *=* *0.56; *P *<* *0.02), HADS anxiety scores (*r *=* *0.44; *P *<* *0.08), body mass index (*r *=* *0.78; *P *<* *0.001) and daytime sleepiness scores (*r *=* *0.48; *P *<* *0.05). No statistical association was found between fatigue symptoms and dopamine or BH4 plasma levels, gender, age, disease duration, daily levodopa dose, years of education and Hoehn and Yard or MOCA. It was also observed a non-significant trend (*P *≤* *0.20) in the association between the PFS-16 scores and the plasma BH4 levels, age and UPDRS-III. As shown by the final model of multiple linear regression (bottom of Table 1), only UPDRS-III score (*B *=* *0.62; CI 95% 0.09 to 1.12; *P *<* *0.02) and blood DOPAC levels (*B* = −2.27; CI −3.57 to −0.97; *P *<* *0.003) remained significant and independently associated with the patients fatigue symptoms. Taken together, the UPDRS-III score and blood DOPAC levels explains 68%, and DOPAC levels alone 34% of the PFS-16 score variation, suggesting a tight association between altered dopamine metabolism and the presence of fatigue symptoms in idiopathic Parkinson’s disease.

## Discussion

Fatigue has been defined as an overwhelming sense of tiredness, lack of energy and feeling of exhaustion.^6^^,^^8^^,^^50^ The clinical diagnosis of fatigue in Parkinson’s disease is complex due to its intrinsic association with other NMS. In a meta-analysis study, Siciliano et al.^51^ demonstrated that whereas fatigue prevalence is not modulated by demographic and motor features or disease progression, it is associated with apathy, anxiety and sleep disturbances.

Although fatigue is commonly reported as a clinical complaint in many neurologic diseases, listed as one of the three most disabling symptoms in at least 58% and 70% of American and Brazilian Parkinson’s disease patients, respectively,^7^^,^^52^^,^^53^ and reported to be experienced on a daily basis for up to 70% of the affected individuals,^7^^,^^8^ it remains an unmet clinical need. Fatigue is also associated with reduced activity levels and physical function, and, frequently, patients need to stop working due to the extreme tiredness.^52^^,^^54^^,^^55^

Despite the enormous impact of fatigue in the quality of life of Parkinson’s disease patients, its pathophysiology has not been fully elucidated. Mechanisms involving exacerbated cytokines production, abnormalities of the hypothalamic–pituitary–adrenal axis function, a pro-inflammatory status and disturbances in basal ganglia circuits have been suggested to underly fatigue in Parkinson’s disease.^6^

Fatigue is classified as either subjective fatigue, involving sensations of tiredness, or fatigability, defined as the inability to maintain motor performance at a desired level, commonly associated with reduced physical activity, like generation of force, or the execution of tasks that require a desired motor performance.^56^^,^^57^ Both forms of fatigue have been defined as independent symptoms of Parkinson’s disease-affected patients. Fatigability appears to be associated with peripheral and central factors, e.g. deficiencies in glycogen stores, and mitochondrial alterations, in muscle and nerves and dysfunctional cortical and subcortical networks, all of which are associated with exercise intolerance.^53^^,^^58^ On the other hand, subjective fatigue has been shown to have a strong association with mood disorders, including depression and anxiety.^52^^,^^59^ Fatigue scales are the most commonly used instruments to clinically score fatigue symptoms due to the lack of quantitative indexes or biochemical biomarkers. According to the International Movement Disorders Society, the scales, including Fatigue Severity Scale (9-items scale), Multidimensional Fatigue Inventory (20-items scale) and PFS (16-items scale), are recommended to assess fatigue symptoms in Parkinson’s disease patients.^56^^,^^60^

In order to understand the involvement of dopamine metabolism in the physiopathology of fatigue in Parkinson’s disease, we used voluntary and forced exercise paradigms to quantify exercise intolerance in animals treated with reserpine. Behaviourally, reserpine administration in rodents induces akinesia and muscular rigidity (catalepsy), which were counterbalanced by levodopa, the gold-standard pharmacological treatment for Parkinson’s disease.^21^^,^^61^ Thus, the reserpine model used in the present study reliably supports the predictive validity to investigate the link between impaired monoamine metabolism and NMS in Parkinson’s disease.

Given the well-defined role of dopamine in the initiation of voluntary movements,^3^^,^^62^ it is plausible that basal ganglia contribute to central fatigue by modulating dopaminergic circuits involved in movement control.^63^ This effect might be worsened by the impaired executive functions induced by dopamine depletion in the prefrontal cortex, leading to inefficient volition, planning, purposive action, and effective performance,^6^^,^^64^ which will ultimately impact on motor behaviour. Here, levodopa restored dopamine and BH4 levels in the striatum of reserpinized mice and attenuated fatigability-like symptoms, characterized by improvement in the distance travelled on the running wheel and by improved performance in the maximal running treadmill test. These data are in agreement with the pioneer findings of Lou et al.^10^ showing reduced physical fatigability in the ‘off’ state of Parkinson’s disease patients under levodopa/carbidopa treatment. The authors reported that Parkinson’s disease patients fatigued during a repetitive motor task and that this drop-off in strength was improved by levodopa.^10^ In addition, Schifitto et al.^65^ demonstrated that levodopa reduced the progression of fatigue scores in Parkinson’s disease patients when compared to individuals receiving placebo, suggesting that dopaminergic pathways are involved in pathogenesis of fatigue. Similarly, it was observed that modafinil (a dopamine transporter blocker) administration during two months was effective in reducing fatigue symptoms, assessed by the Multidimensional Fatigue Inventory scale, and fatigability assessed by finger tapping and intermittent force generation.^11^ However, two other studies that investigated fatigue as secondary outcome measure have found no improvement in fatigue with the use of modafinil, possibly due to the shorter periods of drug treatment.^66^^,^^67^ Methylphenidate (a dopamine transporter blocker) and rosagiline (a monoamine oxidase-B inhibitor) treatments also improved fatigue scores in Parkinson’s disease patients by enhancing the dopaminergic signalling in the striatum.^12^^,^^68^ Altogether, the current evidence generated by our group is in agreement with these previous reports and indicates, at least in part, the involvement of dopaminergic/pterinergic impairment for the induction of fatigue.

The regular practice of physical activity influences exercise tolerability and performance by improving dopamine neurotransmission in brain areas with enriched dopaminergic innervation, including the striatum, the prefrontal cortex and the hippocampus.^58^ One of the most consistent findings in the literature is the observation that brain dopamine content and release is increased during exercise in laboratory rodents.^69^^,^^70^ The seminal study of Bliss and Ailion^71^ showed that immediately after swimming for one hour or running for 30 min, brain dopamine content was significantly increased. In addition, it has been shown that the degree of the exercise-induced dopaminergic enhancement is dependent on the speed, type, and duration of exercise, the gender of the animals, and the appropriate balance between striatal dopamine and serotonin levels.^58^ For example, increased serotonergic neurotransmission characterized by a greater serotonin/dopamine ratio in the striatum has been shown to negatively modulate exercise performance in mice.^58^^,^^72^ In agreement, we observed the lowest striatal serotonin/dopamine ratio in the exercised mice with the highest performance. The inability of reserpine-treated mice in maintaining the exercise performance, even during a submaximal activity, strongly suggests that the impairment of dopamine metabolism contributed to the development of fatigability.

The striatal dopamine metabolic flux is controlled by the rate-limiting enzyme tyrosine hydroxylase, under the absolute dependence on BH4 intracellular levels (Figs 1F and 3A).^13^ Human genetic deficiencies of BH4-synthesizing enzymes are characterized by reduced BH4 levels and a clinical phenotype of Parkinson’s disease-like motor symptoms.^13^^,^^73^^,^^74^ For instance, genetic mutations in an allele of the *GCH1* or mutations in both alleles of sepiapterin reductase (enzymes involved in BH4 synthesis) cause dopa-responsive dystonia due to dopamine deficiency in the brain, and the pharmacological treatment is depicted by the administration of levodopa/carbidopa and 5-hydroxytryptophan plus BH4.^75–77^ In fact, variants in *GCH1* appeared significantly more frequently in idiopathic Parkinson’s disease patients than in controls,^78^ strongly suggesting the participation of BH4 metabolism in Parkinson’s disease physiopathology.

Reduced BH4 levels and activity of the BH4 biosynthetic metabolism have also been demonstrated in the *substantia nigra pars compacta* and striatum of *postmortem* Parkinson’s disease brains.^79^ Indeed, levels of BH4 have been shown to be decreased in the CSF of Parkinson’s disease patients and in rat cerebellar granule neurons treated with MPP^+^, although the molecular mechanisms have not been fully investigated.^80–82^ Furthermore, the degree of reduction of BH4 levels in these reports was similar to the one found in the present study, suggesting that reserpine-induced dopamine depletion was due, at least in part, to compromised striatal BH4 levels. Additionally, our group has recently demonstrated the essential participation of BH4 in energy metabolism, by regulating mitochondrial bioenergetics and oxidative stress in immune cells.^42^ Therefore, it is feasible that BH4 deficiency could also compromise mitochondrial physiology, contributing to cell death in Parkinson’s disease. In addition, our group has also previously reported that, parkin null mice carrying a deletion in exon 3, a genetic model of Parkinson’s disease, display striatal impairments in the *de novo* pathway responsible for maintaining neuronal BH4 levels and an increased number of mitochondria of smaller size with perinuclear clustering during inflammation.^83^

Inflammation and/or oxidative stress may also be responsible for causing a decrease in BH4 levels, since it has been described that BH4 possesses antioxidant properties and prevents from ferroptosis-induced cell death.^42^^,^^82^^,^^84^ However, our data showed no changes in *Gch1* expression, which is transcriptionally controlled by inflammatory mediators,^13^^,^^41^ suggesting that the decreased striatal BH4 levels after reserpine administration did not interfere with *de novo* pathway in the brain. However, it is feasible that the BH4 reduction activated the salvage pathway as a compensatory mechanism, as seen for the increased striatal *Dhfr* expression. Additionally, the restoration of striatal dopamine levels by levodopa appears to also negatively modulate the increased salvage pathway activity, since the metabolic intermediate sepiapterin was significantly increased in the blood. Our group has recently demonstrated that the use of sepiapterin reductase inhibitors, also produce increased levels of sepiapterin in tissues and biological fluids, allowing us to propose this pteridine as a biomarker for a compromised synthesis of BH4 in the brain.^41^^,^^43^ This opens future investigations to measure sepiapterin levels in the blood of Parkinson’s disease patients.

DOPAC is the main neuronal metabolite of dopamine metabolism and its formation is catalysed by two enzymatic steps coordinated by monoamine oxidase and aldehyde dehydrogenase.^49^ Several reports have shown low CSF dopamine and DOPAC levels in Parkinson’s disease patients,^85^^,^^86^ which is thought to reflect the loss of nigrostriatal dopaminergic neurons.^85–88^ Conversely, treatment with levodopa has shown to increase CSF levels of catecholamine and its metabolites;^88^^,^^89^ however, studies measuring this pathway in the blood are scarce. Here, we found that lower blood DOPAC levels inversely and independently correlated with higher fatigue scores in idiopathic Parkinson’s disease patients under chronic treatment with levodopa. This blood biomarker is partly derived from intraneuronal deamination of dopamine in sympathetic nerves;^85^ therefore, it is feasible that the lower levels observed in the patients’ plasma denote impaired brain dopamine metabolism. Blood DOPAC levels also positively correlated with the progression of the motor disease, indicating that as the disease progresses there is a need for higher levodopa doses.

In addition, fatigue has been shown to be associated with other common NMS of Parkinson’s disease, such as depression.^90–92^ Indeed, our results showed a positive association between fatigue symptoms and HADS depression and anxiety scores. However, we did not identify compromised serotoninergic metabolism in the striatum of the mice, suggesting depression might not be involved in the induction of fatigue in our experimental model. In agreement, a prospective longitudinal study showed that fatigue can occur independently of depression, and that non-depressed Parkinson’s disease patients may exhibit more fatigue than depressed Parkinson’s disease patients.^93^ Moreover, the lack of association between fatigue and disease duration suggests that, in a large proportion of patients with Parkinson’s disease, fatigue is present in the early phase of disease and tends to persist over time. Therefore, as previously suggested, fatigue is not likely to emerge as a result of disease progression or comorbidity with depression or cognitive dysfunction.^51^ Finally, the positive correlation observed between fatigue and body mass index might also argue that obese individuals presented worse fatigue scores due a hypokinetic state promoted by adiposity. For instance, the weight of excess adiposity restricts joint and muscle mobility and increases joint pain, which may make it more difficult for people to move.^94^ However, body mass index alone did not explain the variation of the fatigue symptoms score in the Parkinson’s disease patients (Table 1).

Altogether, the data presented here show that dopamine and BH4 metabolisms are impaired in the striatum of reserpine-treated mice with exercise intolerance. The positive regulation of the salvage pathway might represent a central compensatory mechanism to re-establish the appropriate dopaminergic transmission. Although it has previously been reported that the severity of fatigability does not correlate with fatigue in Parkinson’s disease patients,^95^ to our knowledge, this study provides the first evidence that levodopa may attenuate fatigability-like symptoms in reserpinized mice. Thus, this study has the potential to open new avenues for further studies aimed to better understand the relationship between fatigability and fatigue. Moreover, if confirmed in larger cohorts from different ethnical groups, the current findings may be useful as a fatigue quantitative index in Parkinson’s disease patients, since DOPAC levels represents the main neuronal dopamine metabolite in plasma.

## Conclusions

The data presented suggest that (i) the striatal deficiency of dopamine and BH4 may lead to performance fatigability in the reserpine mouse model of Parkinson’s disease; (ii) the deficiency of dopamine levels in the striatum may be rescued by increasing the BH4 salvage pathway alleviating fatigability-like symptoms; (iii) nociceptive, cardiovascular, metabolic or emotional factors did not seem to influence directly the fatigability symptoms induced by reserpine; and (iv) reduced blood DOPAC levels might result in a quantitative biomarker for fatigue progression in idiopathic Parkinson’s disease patients under levodopa therapy if confirmed in larger cohorts. Therefore, it can be concluded that fatigability-like symptoms might be caused by impaired central dopaminergic transmission provoked by deficiency of dopamine and/or BH4 metabolisms, and peripherally assessed by following DOPAC plasma levels.

## Supplementary material

Supplementary material is available at *Brain Communications* online.

## Supplementary Material

fcab116_Supplementary_Data

## Abbreviations

BH4 =: tetrahydrobiopterin
DOPAC =: 3,4-dihydroxyphenyilacetic acid
HADS =: hospital anxiety and depression scale
HPLC =: high performance liquid chromatography
l-DOPA =: l-3,4-dihydroxyphenylalanine
MOCA =: Montreal Cognitive Assessment
NMS =: non-motor symptoms
PFS =: Parkinson fatigue scale
UPDRS =: unified Parkinson’s disease rating scale
