# Supplementary material for: Impaired dopamine metabolism is linked to fatigability in mice and fatigue in Parkinson’s disease patients
Source: Brain Commun. 2021 Jun 8;3(3):fcab116. doi: 10.1093/braincomms/fcab116 (PMC8374980; doi:10.1093/braincomms/fcab116)
Supplement: fcab116_Supplementary_Data [file fcab116_Supplementary_Data.zip › Supplementary_material.docx]

**Supplementary material**

| **Supplementary Table1:** Socio-demographic, clinical and laboratorial characteristics of Parkinson’s disease patients | | | | | | |
| --- | --- | --- | --- | --- | --- | --- |
| **Socio-demographic, clinical and laboratorial variables** |  | Parkinson’s disease patients  (*n*= 14-18)  **n (%) or mean (SD)** |  | Controls  (*n*= 17-18)  **n (%) or mean (SD)** |  | *P*  level |
| Gender  male  female |  | 04 (22.2)  14 (77.8) |  | 09 (50.0)  09 (50.0) |  | 0.16 |
| Age (years) |  | 61.6 (9.7) |  | 61.65 (10.0) |  | 0.93 |
| Disease duration (years) |  | 14.4 (5.5) |  | N.A. |  |  |
| UPDRS-III score |  | 45.1 (19.2) |  | N.A. |  |  |
| Hoehn & Yard score |  | 4.4 (1.5) |  | N.A. |  |  |
| Daily levodopa dose (mg/kg) |  | 14.1 (8.7) |  | N.A. |  |  |
| MOCA score |  | 17.9 (6.8) |  | N.A. |  |  |
| Education (years) |  | 6.0 (4.7) |  | N.A. |  |  |
| HADS depression score |  | 8.9 (5.0) |  | N.A. |  |  |
| HADS anxiety score |  | 8.0 (4.3) |  | N.A. |  |  |
| Body mass index (kg/m^2^) |  | 27 (4.9) |  | N.A. |  |  |
| PFS-16 score |  | 51.8 (19.6) |  | N.A. |  |  |
| **Blood parameters** |  |  |  |  |  |  |
| DOPAC levels (nmol/L) |  | 6.8 (6.1) (n =14) |  | N.D. (*n*= 17) |  | < 0.0001 |
| HADS = hospital anxiety and depression scale; MOCA = Montreal cognitive assessment; PSF-16= Parkinson’s Disease Fatigue Scale; UPDRS-III = unified Parkinson’s disease rating scale | | | | | | |

**Drugs**

All drugs were obtained from Sigma Chemical (MO, USA). Drug treatments or vehicle injection were performed by the intraperitoneal (i.p.) route in a volume of 10.0 mL/kg. Reserpine (methyl reserpate 3,4,5-trimethoxybenzoic acid ester; 1 mg/kg) was dissolved in 0.1% acetic acid (vehicle). Levodopa (L-3,4-dihydroxyphenyl-alanine methyl ester hydrochloride; 100 mg/kg) and benserazide (benserazide hydrochloride; 50 mg/kg) were dissolved in saline solution, containing 0.1% sodium metabisulfite. The selected doses of levodopa and benserazide were based on previous literature and on pilot experiments carried out in our laboratory^1^.

**Exhaustion test protocol**

All mice were transported and habituated to the experimental room 1 h before the test. The measurements were carried out during the nocturnal phase of the animal cycle (between 8:00 p.m. and 10:00 p.m.) on a treadmill adapted for small rodents. The animals were adapted for five days to the treadmill (10 m/min; 10 min), and 48 h later the incremental loading test was performed. The running protocol started at 16 m/min, with 2 m/min increment every 3 min, at 9% treadmill grade until animal exhaustion. Immediately afterwards, 25 µL of blood was taken from the tail vein for lactate measurements. Time to exhaustion (min) and workload (N.m) were considered indexes of exercise performance. Exercise workload (N.m) was calculated as follows: (body mass in kg) x (9.81 m/s^2^) x (treadmill speed in m/s) x (time of exercise in min) x (treadmill inclination in %)^2^.

**Forced swimming test**

Depressive-like symptoms were assessed on the forced swimming test. Animals were individually forced to swim in a cylinder (Ø 16 cm, 30 cm high) filled with water at 25 ± 1°C (15 cm high)^3,4^. Forced swimming test was monitored by using a video tracking system with the camera positioned above the apparatus. Body immobility was scored during 5 min using the behavioral tracking software ANY- Maze^TM^ (IL, USA) in an adjacent room by an experienced experimenter.

**Splash test**

The self-care and motivational behaviors were assessed by measuring the time spent in grooming behavior, after mice being squirted with 1 mL of a 10% sucrose solution on the dorsal coat as previously reported^5^. The time spent grooming was recorded for a period of 5 min in an observation device (Crimson Scientific, MA, USA) which consisted of an acrylic box with black walls (20 cm x 20 cm x 20 cm), a black lid on the top, a glass floor, and lit by red light to reduce stress. A camera located below the box pointing up through the glass floor was used to observe the animal behavior (videos 1-2). The amount of grooming was scored after the experiment had been completed from video, by an observer blind to treatment using a stop watch.

**Von Frey test**

The threshold for mechanical nociception was determined with a graded series of eight von Frey filaments that produced a bending force of 0.16, 0.4, 0.6, 1, 1.4 and 2 g, respectively. Each filament was tested 10 times in increasing order starting with the filament producing the lowest force. Von Frey filaments were applied at least 3 s after the mice had returned to their initial resting state. For baseline mechanical sensitivity test all filaments were applied and the number of withdrawals was recorded. For tactile allodynia: the minimal force filament for which animals presented either a brisk paw withdrawal and/or an escape attempt in response to at least 5 of the 10 stimulations determined the mechanical response threshold^6,7^.

**ECG profile**

The peripheral effect of the administered drugs on the heart's rhythm and activity was determined by assessing the ECG. Conscious and unrestrained mice were placed onto the platform of the ECGenie system (Mouse Specifics, MA, USA) and allowed 2 to 3 min to acclimatize before ECG recording. Once a continuous period of 5 s of ECG signals was recorded the animal was removed back to its home cage. The recording rate interval and heart rate were calculated automatically by the software.

**Quantitation of levodopa, dopamine, 3,4-dihydroxyphenyilacetic acid (DOPAC) and serotonin levels**

Monoamines and their metabolites were measured in the striatum and/or the blood by high performance liquid chromatography (HPLC). Five to six Swiss mice from each experimental group were euthanized by decapitation and their brains were removed immediately and had the striatum dissected, weighed, and immediately frozen and stored at -80°C. The brain tissue was sonicated and centrifuged at 16,000 x *g* for 10 min at 4ºC, in chilled 0.1 M perchloric acid (1:10, v/v). Plasma samples from Parkinson’s disease patients were precipitated by the addition of one volume (1:1, v/v) of 0.5 M perchloric acid containing 0.02% sodium metabisulfite and centrifuged at 16,000 x *g* for 10 min at 4ºC. Monoamines and their metabolites present in the supernatants were assessed by HPLC (Alliance e2695, Waters, MA, USA), with electrochemical detection (Waters 2465, Waters, MA, USA) with the voltage set at +400 mV. The temperature of the column compartment was set at 35°C. Twenty microliters of supernatant were analyzed in a 150 x 2.0 mm, 4 µm, C18 column (Synergi Hydro, CA, USA) with a mobile phase containing 90 mM sodium phosphate, 50 mM citric acid, 1.7 mM sodium 1-heptane-sulfonate, 50 µM ethylenediaminetetraacetic acid, 10% acetonitrile, pH 3.0, with a flow of 0.25 mL/min^2^. L-DOPA, dopamine, DOPAC and serotonin levels in the supernatants were calculated as ηg/mg protein. Blood DOPAC levels were calculated as nmol/L.

**Quantitation of BH4 levels**

Striatal BH4 levels were determined by HPLC coupled with electrochemical detection as previously described with some modifications^7,8^. Briefly, brain tissue was homogenized in 100 μL of 60 mM potassium phosphate buffer and centrifuged at 10,000 × *g* for 10 min at 4°C. Brain homogenates were precipitated by the addition of one volume (1:1, v/v) of 0.1 M hydrochloric acid. Afterwards, samples were centrifuged at 16,000 × *g* for 10 min at 4°C, and 20 μL of supernatant were transferred to an HPLC vial for further analysis. The HPLC measurement of BH4 was carried out in a HPLC (Alliance e2695, Waters, MA, USA) by using a Waters Atlantis dC18, reverse phase column (4.6 × 250 mm; 5 μm particle), with a flow rate set at 0.7 mL/min and an isocratic elution of 6.5 mM NaH_2_PO_4_, 6 mM citric acid, 1 mM sodium octyl sulfate, 2.5 mM diethylenetriaminepentaacetic acid, 160 μM dithiothreitol and 12% acetonitrile, pH 3.0. The temperature of the column compartment was set at 35°C. The identification and quantification of BH4 was performed by coupling to the HPLC an electrochemical detector (module 2465, Waters, MA, USA) with the voltage set at +450 mV. The results were expressed as pmol/mg protein.

**Quantitation of sepiapterin levels**

Serum samples were precipitated by adding one volume of 5% trichloroacetic acid and centrifuged at 16,000 × *ɡ* for 10 min at 4ºC. Twenty microliters of supernatant were transferred to a HPLC vial for analysis. The HPLC analysis was performed in an Alliance e2695system (Waters, MA, USA) by using a Waters Atlantis dC-18 5 μm reverse phase column (4.6 × 250 mm), with a flow rate set at 0.7 mL/min and an isocratic elution of 85% phosphate buffer 15 mM and 15% acetonitrile, pH 6.4. The temperature of the column compartment was set at 35°C. The identification and quantification of sepiapterin were performed by coupling to the HPLC system a multi-wavelength fluorescence detector (module 2475, Waters, MA, USA) with excitation wavelength of 425 nm and emission of 530 nm^9^. The results were expressed as nmol/L.

**Gene expression analysis by quantitative real-time PCR**

Total RNA was isolated from striatum samples by using the TRIzol®/chloroform/isopropanol method as previously described by our group^10^. The quantity and purity of extracted RNA was estimated by using the spectrophotometer apparatus NanoDrop, at 260 nm and 280 nm. The cDNA (3 µg of total RNA per reaction) was synthesized by using the reverse transcription kit “M-MLV Reverse Transcriptase” (Sigma, MO, USA), according to the instructions recommended by the manufacturer. qPCR was performed by using SYBR Green Master Mix (Applied Biosystems, CA, USA) and specific primers (Table 1) for each gene. The primers were designed using the "BLAST" available at: http://blast.ncbi.nlm.nih.gov/Blast.cgi according exons specific for each gene. Reactions were performed in the ABI PRISM 7900HT equipment (Applied Biosystems, CA, USA) in the Multiuser Laboratory for Biological Studies (LAMEB, UFSC, Brazil). The results were analyzed using the Sequence Detection System software version 2.4. The critical comparative threshold method 2^-ΔCt^ was used to calculate the relative number of transcripts in the samples. In this method, the average Ct gene of interest is subtracted from the average Ct internal control (β-actin), resulting in a ΔCt. To calculate gene expression, the ΔCt value obtained is replaced in 2^-ΔCt^ formula. The obtained final numbers are presented as the ratio between the expression of the gene of interest relative to the internal control gene. The results were expressed as mean ± S.E.M. of 3 independent animals performed in triplicate.

| **Supplementary Table 2**. Primers used for gene expression analysis | | |
| --- | --- | --- |
| Gene | Forward sequence | Reverse sequence |
| *Actb* | 5’ GCGTCCACCCGCGAGTACAAC 3’ | 5’ CGACGACGAGCGCAGCGATA 3’ |
| *Gch1* | 5’ TGAGCCCCAGTCCGGGTGAC 3’ | 5’ GTGCTAACAAGCGCTGCGGC 3’ |
| *Pts* | 5’ GTCCTTCAGCGCGAGCCACC 3’ | 5’ CCCGTGTGAGGCCCTGGTGT 3’ |
| *Spr* | 5’ CCGAGTGTGCGGGTGCTGAG 3’ | 5’ CCAGCGCCCCATCCGACTTC 3’ |
| *Dhfr* | 5’AAAGTGGACATGGTCTGGGTA 3’ | 5’CTGGCTGATTCATGGCTTC 3’ |
| *Qdpr* | 5’GCCAGCGTGGTTGTTAAGAT 3’ | 5’ AAGAGGCCTCCTTCCTTCAG 3’ |

*Act* = β-actin; *Dhfr* = dihydrofolate reductase; *Gch1* = guanosine triphosphate cyclohydrolase I; *Pts* = 6-pyruvoyl tetrahydropterin synthase; *Qdpr* = dihydropteridin reductase; *Spr* = sepiapterin reductase

**Cellular viability assay**

L6 myotubes were seeded at a density of 1 x 10^4^ per well in 96-well plates in appropriate medium. MTT (3[4,5-dimethylthiazol-2-yl]-2,5-diphenyltetrazolium bromide) assay was used to evaluate cellular viability. Active dehydrogenases cleave and reduce the soluble yellow MTT dye into the insoluble purple formazan^11^. Cells were incubated with 10 μM reserpine and/or 20 μM rotenone (positive control) for 3 h in a 5% CO_2_/95% O_2_, humidified atmosphere, at 37ºC. At the end of the incubation period, MTT test was performed. The formazan formation was spectrophotometrically assayed at 570 nm. Results are indicated as percentage of controls.

**Measurement of mitochondrial respiration**

L6 myotubes were seeded at a density of 2 x10^5^ per well in 6-well plates in appropriate medium. Non-permeabilized cells were suspended in appropriate medium without fetal bovine serum at a cell concentration of 300,000 cells/mL. Oxygen consumption was measured in L6 myotubes at 37°C by high-resolution respirometry using the Oroboros® oxygraph with chamber volumes set at 2 mL, as previously described by our group^12^. DatLab software (Oroboros Instruments, Innsbruck, Austria) was used for data acquisition and analysis. The experimental regime was started by measuring *basal respiration* without the addition of substrates or effectors. After observing a steady-state respiratory flux (about 15 to 30 min; green bar Fig. 4I), a titration with incremental doses of reserpine (0.005; 0.05; 0.5 and 5 ηg/mL) or vehicle (acetic acid 0.1%) was performed. Then, the ATP synthase activity was inhibited with 1 μg/mL oligomycin, measurement defined as *leak state* (non-phosphorylating mitochondrial resting state where oxygen flux is maintained mainly to compensate for the proton leak at a high chemiosmotic potential), followed by uncoupling of oxidative phosphorylation by stepwise titration with FCCP (carbonyl cyanide p-trifluoromethoxyphenylhydrazone) up to the *maximal mitochondrial electron transfer activity*, defined as ETS. Finally, respiration was inhibited by the sequential addition of 0.5 μM rotenone (inhibitor of complex I activity) and 2.5 μM antimycin A (inhibitor of complex III activity), in order to measure the *residual oxygen consumption*, stated as ROX. The contribution of ATP synthase activity to the basal oxygen consumption was also calculated after oligomycin exposure, and it was expressed in % of controls.

**Supplementary references:**

1. Singh A, Kulkarni SK. Nitecapone and selegiline as effective adjuncts to L-DOPA in reserpine-induced catatonia in mice. *Methods Find Exp Clin Pharmacol*. 2002;24(1):23-29. doi:10.1358/mf.2002.24.1.677124

2. Scheffer D da L, Ghisoni K, Aguiar AS, Latini A. Moderate running exercise prevents excessive immune system activation. *Physiol Behav*. 2019;204:248-255. doi:10.1016/j.physbeh.2019.02.023

3. Porsolt RD, Bertin A, Jalfre M. Behavioral despair in mice: a primary screening test for antidepressants. *Arch Int Pharmacodyn Ther*. 1977;229(2):327-336. http://www.ncbi.nlm.nih.gov/pubmed/596982. Accessed December 20, 2017.

4. Godbout JP, Moreau M, Lestage J, et al. Aging exacerbates depressive-like behavior in mice in response to activation of the peripheral innate immune system. *Neuropsychopharmacology*. 2008;33(10):2341-2351. doi:10.1038/sj.npp.1301649

5. Nollet M, Guisquet A-M Le, Belzung C. Models of Depression: Unpredictable Chronic Mild Stress in Mice. In: *Current Protocols in Pharmacology*. Vol Chapter 5. Hoboken, NJ, USA: John Wiley & Sons, Inc.; 2013:Unit 5.65. doi:10.1002/0471141755.ph0565s61

6. Dixon WJ. Efficient Analysis of Experimental Observations. *Annu Rev Pharmacol Toxicol*. 1980;20(1):441-462. doi:10.1146/annurev.pa.20.040180.002301

7. Latremoliere A, Latini A, Andrews N, et al. Reduction of Neuropathic and Inflammatory Pain through Inhibition of the Tetrahydrobiopterin Pathway. *Neuron*. 2015;86(6):1393-1406. doi:10.1016/j.neuron.2015.05.033

8. Cronin SJF, Seehus C, Weidinger A, et al. The metabolite BH4 controls T cell proliferation in autoimmunity and cancer. *Nature*. 2018;563(7732):564-568. doi:10.1038/s41586-018-0701-2

9. Fujita M, da Luz Scheffer D, Lenfers Turnes B, et al. Sepiapterin reductase inhibition selectively reduces inflammatory joint pain and increases urinary sepiapterin. *Arthritis Rheumatol*. 2019;72(1):57-66. doi:10.1002/art.41060

10. Latini A, de Bortoli da Silva L, da Luz Scheffer D, et al. Tetrahydrobiopterin improves hippocampal nitric oxide-linked long-term memory. *Mol Genet Metab*. 2018;125:104-111. doi:10.1016/j.ymgme.2018.06.003

11. Mosmann T. Rapid colorimetric assay for cellular growth and survival: application to proliferation and cytotoxicity assays. *J Immunol Methods*. 1983;65(1-2):55-63. http://www.ncbi.nlm.nih.gov/pubmed/6606682. Accessed December 20, 2017.

12. Remor AP, de Matos FJ, Ghisoni K, et al. Differential effects of insulin on peripheral diabetes-related changes in mitochondrial bioenergetics: Involvement of advanced glycosylated end products. *Biochim Biophys Acta - Mol Basis Dis*. 2011;1812(11):1460-1471. doi:10.1016/j.bbadis.2011.06.017
